# Supplementary material for: Molecular Diversity of Giardia duodenalis, Cryptosporidium spp., and Blastocystis sp. in Symptomatic and Asymptomatic Schoolchildren in Zambézia Province (Mozambique)
Source: Pathogens. 2021 Feb 24;10(3):255. doi: 10.3390/pathogens10030255 (PMC7996272; doi:10.3390/pathogens10030255)
Supplement: Supplementary file 1 [file pathogens-10-00255-s001.zip › supplementary 2/Figure S1_Pathogens_2021_Muadica_et_al..docx]

**Figure S1.** Phylogenetic relationships among Giardia duodenalis assemblages and sub-assemblages identified in infected symptomatic and asymptomatic children in the Zambézia province, Mozambique. The analysis was conducted by a neighbor-joining method of a 502-bp fragment (corresponding to position 101–602 of reference sequence AY072727) of the bg gene sequence. Genetic distances were calculated using the Kimura two-parameter model. Green filled squares represent sequences generated in the present study. Purple filled dots represent reference sequences. Bootstrap values lower than 50% are not displayed. Because bg is a Giardia-specific protein, no outgroup taxon was used to root the tree.
