# Supplementary material for: Molecular Diversity of Giardia duodenalis, Cryptosporidium spp., and Blastocystis sp. in Symptomatic and Asymptomatic Schoolchildren in Zambézia Province (Mozambique)
Source: Pathogens. 2021 Feb 24;10(3):255. doi: 10.3390/pathogens10030255 (PMC7996272; doi:10.3390/pathogens10030255)
Supplement: Supplementary file 1 [file pathogens-10-00255-s001.zip › supplementary 2/Table S1_Pathogens_2021_Muadica_et_al..docx]

**Table S1.** Diversity, frequency, and main molecular features of *Giardia duodenalis* sequences at the *gdh* locus in infected symptomatic and asymptomatic children in the Zambézia province, Mozambique. GenBank accession numbers are provided. Superscript numbers identify single nucleotide polymorphisms involving amino acid change.

| **Assemblage** | **Sub-Assemblage** | **No. of isolates** | **Reference sequence** | **Stretch** | **Single Nucleotide Polymorphisms** | **GenBank ID** |
| --- | --- | --- | --- | --- | --- | --- |
| A | AII | 4 | L40510 | 68–497 | Ninguno | MW508361 |
| B | BIII | 1 | AF069059 | 40–460 | C64Y^1^, C87Y, C99Y, T147C, G150R, C309Y, C336Y, G402R, T456Y | MW508362 |
|  |  | 1 | AF069059 | 40–460 | C69Y, C99Y, T147Y, G150R, C309Y, C336Y, T456Y | MW508363 |
|  |  | 1 | AF069059 | 40–455 | C69Y, T147Y, C336Y, G402R, T426Y | MW508364 |
|  |  | 1 | AF069059 | 40–460 | T147Y, T219C, C330Y, G381A, G402A | MW508365 |
|  |  | 1 | AF069059 | 40–460 | C309T | MW508366 |
|  | BIV | 1 | L40508 | 76–491 | C111Y, T183C, C345Y, T366C, T387C, C396Y, C423Y, A438R | MW508367 |
|  |  | 1 | L40508 | 76–491 | T135C, T183C | MW508368 |
|  |  | 1 | L40508 | 76–496 | T183C, C291Y, T366Y, T387C, C423Y, C432Y | MW508369 |
|  |  | 1 | L40508 | 76–496 | T183C, T336C, T387C, C396T, C423T | MW508370 |
|  |  | 1 | L40508 | 76–491 | T183Y, C345Y, T366Y, T387C | MW508371 |
|  |  | 1 | L40508 | 76–496 | T183C, T387C | MW508372 |
|  |  | 2 | L40508 | 76–480 | T183C, T387C, C396T, C423T | MW508373 |
|  |  | 1 | L40508 | 76–422 | G276A, C327T | MW508374 |
|  | BIII/BIV | 1 | L40508 | 76–491 | G84R, C126Y, T135Y, C255Y, C273Y, T366Y, T387C, C423Y, C432Y, A438R | MW508375 |
|  |  | 1 | L40508 | 76–491 | G93R, C123Y, T135Y, T183Y, C255Y, C273Y, C345Y, T366Y, C372Y, T387Y, A438R | MW508376 |
|  |  | 1 | L40508 | 76–491 | C105T, T135Y, T183Y, C255Y, C273T, C345Y, T366C, C372Y, T387C, A438R | MW508377 |
|  |  | 1 | L40508 | 76–496 | C105Y, T148Y^2^, T183C, G186R, C255Y, C273Y, C345Y, T366Y, C372Y, G384R, T387C, C396Y, C432Y, A438R | MW508378 |
|  |  | 1 | L40508 | 76–491 | T135Y, C159Y, T183Y, C255Y, C273Y, C300Y, C345Y, T366Y, C372Y, T387C, C396Y, A438R | MW508379 |
|  |  | 1 | L40508 | 76–491 | T135Y, A166R^3^, T183C, G186R, C240Y, C255Y, C273Y, T366Y, C372Y, T387C, C396Y, A438R | MW508380 |
|  |  | 1 | L40508 | 76–491 | T135Y, C168Y, T183C, G186R, C255Y, G261R, C273Y, T312Y, C345Y, T366Y, C372Y, T387C, G408R, A438R, G442R^4^ | MW508381 |
|  |  | 1 | L40508 | 76–491 | T135Y, C168Y, T183C, G186R, C255Y, G261R, C273T, T312Y, C345Y, T366Y, C372Y, T387C, G417R, A438R | MW508382 |
|  |  | 1 | L40508 | 76–491 | T135Y, T183Y, G186R, T188Y^5^, C255Y, C273Y, C345Y, A362R^6^, T366Y, C372Y, T387C, C423Y, A438R, A450R, G451R^7^ | MW508383 |
|  |  | 1 | L40508 | 76–496 | T135Y, T183C, G186R, C255Y, C273Y, G294R, C345Y, T366Y, T387C, G417R, A438R | MW508384 |
|  |  | 1 | L40508 | 76–436 | T135Y, T183C, A187R, C255Y, T387C, C423T | MW508385 |
|  |  | 1 | L40508 | 76–491 | T135Y, T183Y, C255Y, C273Y, T366C, T387Y, A438R | MW508386 |
|  |  | 1 | L40508 | 76–491 | T135Y, C255Y, C273Y, C345Y, T366Y, C372Y, T387Y, A438R | MW508387 |
|  |  | 1 | L40508 | 76–491 | T135Y, T366Y, T387C, C423Y, A438R | MW508388 |
|  |  | 1 | L40508 | 76–496 | T135Y, T366Y, T387C, C423Y, A438R, G442R | MW508389 |
|  |  | 1 | L40508 | 76–491 | G156R, T183C, G186R, C255Y, C273T, T312Y, C345Y, T366Y, C372Y, T387C, A438R | MW508390 |
|  |  | 1 | L40508 | 76–491 | T183Y, G186R, C192Y, C255Y, C273Y, G294R, C345Y, T366Y, C372Y, C387Y, A438R | MW508391 |
|  |  | 1 | L40508 | 76–491 | T183Y, G186R, G201R, C255Y, C273Y, T312Y, C345Y, T366Y, C372Y, C387C, C432Y, A438R | MW508392 |
|  |  | 1 | L40508 | 76–491 | T183Y, G186R, G201R, C255Y, C273Y, T312Y, C345Y, T366Y, C372Y, T387C, C432Y, A438R | MW508393 |
|  |  | 1 | L40508 | 76–491 | T183C, G186R, C255Y, C273Y, C345T, T366Y, C372Y, T387C, C396Y, C423Y, A438R | MW508394 |

^1^ If T, pL22F.

^2^ If C, pF50L.

^3^ If G, pN56D.

^4^ If A, pE148K.

^5^ If C, pM63T.

^6^ If G, pQ121R.

^7^ If A, pG151R.
