# Supplementary material for: Molecular Diversity of Giardia duodenalis, Cryptosporidium spp., and Blastocystis sp. in Symptomatic and Asymptomatic Schoolchildren in Zambézia Province (Mozambique)
Source: Pathogens. 2021 Feb 24;10(3):255. doi: 10.3390/pathogens10030255 (PMC7996272; doi:10.3390/pathogens10030255)
Supplement: Supplementary file 1 [file pathogens-10-00255-s001.zip › supplementary 2/Table S2_Pathogens_2021_Muadica_et_al.docx]

**Table S2.** Diversity, frequency, and main molecular features of *Giardia duodenalis* sequences at the *bg* locus in infected symptomatic and asymptomatic children in the Zambézia province, Mozambique. GenBank accession numbers are provided. Superscript numbers identify single nucleotide polymorphisms involving amino acid change.

| **Assemblage** | **Sub-Assemblage** | **No. of isolates** | **Reference sequence** | **Stretch** | **Single Nucleotide Polymorphisms** | **GenBank ID** |
| --- | --- | --- | --- | --- | --- | --- |
| A | AII | 1 | AY072723 | 97–593 | None | MW508395 |
| B | – | 4 | AY072727 | 100-753 | None | MW508396 |
|  | – | 1 | AY072727 | 94-592 | G165Y, A183R, C309T | MW508397 |
|  | – | 1 | AY072727 | 102-590 | C165T, A228W^1^, C309T, A323W^2^, A497R^3^ | MW508398 |
|  | – | 2 | AY072727 | 102-592 | C165T, C309T | MW508399 |
|  | – | 2 | AY072727 | 102-593 | A183G | MW508400 |
|  | – | 1 | AY072727 | 102-590 | A183G, G261R, C309T | MW508401 |
|  | – | 4 | AY072727 | 102-593 | A183G, C309T, T519C, C564T | MW508402 |
|  | – | 1 | AY072727 | 102-592 | A183G, C309Y, C333Y, T519Y, C564Y | MW508403 |
|  | – | 1 | AY072727 | 102-593 | A183G, C309T, T519Y, C564Y | MW508404 |
|  | – | 2 | AY072727 | 102-590 | A183R, C309Y, T519Y, C564Y | MW508405 |
|  | – | 1 | AY072727 | 102-590 | A183G, C450A^4^ | MW508406 |
|  | – | 1 | AY072727 | 99-590 | C204A, C309T | MW508407 |
|  | – | 1 | AY072727 | 98-592 | T519C, C564T | MW508408 |
|  | – | 1 | AY072727 | 98-590 | C564Y | MW508409 |
|  | – | 1 | AY072727 | 103-590 | A575R^5^ | MW508410 |

^1^ If T, pE76D.

^2^ If T, pD108V.

^3^ If G, pE166G.

^4^ pN150K.

^5^ If G, pE192G.
