# Supplementary material for: Molecular Diversity of Giardia duodenalis, Cryptosporidium spp., and Blastocystis sp. in Symptomatic and Asymptomatic Schoolchildren in Zambézia Province (Mozambique)
Source: Pathogens. 2021 Feb 24;10(3):255. doi: 10.3390/pathogens10030255 (PMC7996272; doi:10.3390/pathogens10030255)
Supplement: Supplementary file 1 [file pathogens-10-00255-s001.zip › supplementary 2/Table S3_Pathogens_2021_Muadica_et_al.docx]

**Table S3.** Diversity, frequency, and main molecular features of *Giardia duodenalis* sequences at the *tpi* locus in infected symptomatic and asymptomatic children in the Zambézia province, Mozambique. GenBank accession numbers are provided. Superscript numbers identify single nucleotide polymorphisms involving amino acid change.

| **Assemblage** | **Sub-Assemblage** | **No. of isolates** | **Reference sequence** | **Stretch** | **Single Nucleotide Polymorphisms** | **GenBank ID** |
| --- | --- | --- | --- | --- | --- | --- |
| A | AII | 1 | U57897 | 276–805 | C287G | MW556751 |
|  |  | 1 | U57897 | 276–804 | C287G, A291W^1^ | MW556752 |
|  |  | 1 | U57897 | – | Unknown^2^ | ‒ |
| B | BIII | 1 | AF069561 | 1–431 | C34T^3^, C43T^4^, C108T, C337T | MW556753 |
|  |  | 1 | AF069561 | 1–456 | C34Y^5^, G105R, C108Y, C141Y, G189R, C324Y, A426R, C447Y | MW556754 |
|  |  | 1 | AF069561 | 1–456 | C34Y^5^, C108Y, C141Y, G189R | MW556755 |
|  |  | 1 | AF069561 | 1–456 | C34Y^5^, C108Y, C141Y, G189R, C214Y^6^, A240R, T276Y, A426R | MW556756 |
|  |  | 1 | AF069561 | 1–456 | C34T^3^, C108T, T291C | MW556757 |
|  |  | 1 | AF069561 | 1–456 | C34Y^5^, C108Y, T291Y | MW556758 |
|  |  | 1 | AF069561 | 1–456 | C108T, C111T, C141T, C148T,354delC^7^ | MW556759 |
|  |  | 1 | AF069561 | 16–456 | C92Y^8^, C108Y, C141Y,A426R | MW556760 |
|  |  | 1 | AF069561 | 1–426 | G153A | MW556761 |
|  |  | 1 | AF069561 | 1–456 | C214T^9^, A223G^10^, A426G | MW556762 |
|  | BIII/BIV | 1 | AF069560 | 1–449 | A5G, T11Y, T57C^11^, T131Y, T134Y, A176R, A395G | MW556763 |
|  |  | 1 | AF069560 | 24–479 | T57C^11^, A70R^12^, A176R, C237Y^13^, T299Y, C323Y, A395G | MW556764 |

^1^ If T, pN12Y.

^2^ Sequence of suboptimal quality to determine the presence of single nucleotide polymorphisms.

^3^ pH12Y.

^4^ pP15S.

^5^ If T, pH12Y.

^6^ If T, pH72Y.

^7^ del: nucleotide deletion associated to a stop codon.

^8^ If T, pT31I.

^9^ pH72Y.

^10^ pI75V.

^11^ pY19H.

^12^ If G, pE23G.

^13^ If T, pH79Y.
