# Supplementary material for: Molecular Diversity of Giardia duodenalis, Cryptosporidium spp., and Blastocystis sp. in Symptomatic and Asymptomatic Schoolchildren in Zambézia Province (Mozambique)
Source: Pathogens. 2021 Feb 24;10(3):255. doi: 10.3390/pathogens10030255 (PMC7996272; doi:10.3390/pathogens10030255)
Supplement: Supplementary file 1 [file pathogens-10-00255-s001.zip › supplementary 2/Table S4_Pathogens_2021_Muadica_et_al.docx]

**Table S4.** Oligonucleotides used for the molecular identification and/or characterization of *Giardia duodenalis*, *Cryptosporidium* spp., and *Blastocystis* sp. in the present study.

| **Target organism** | **Locus** | **Oligonucleotide** | **Sequence (5´–3´)** | **Reference** |
| --- | --- | --- | --- | --- |
| *Giardia duodenalis* | *ssu* rRNA | Probe | FAM–CCCGCGGCGGTCCCTGCTAG–BHQ1 | [55] |
|  |  | Gd-80F | GACGGCTCAGGACAACGGTT | [55] |
|  |  | Gd-127R | TTGCCAGCGGTGTCCG | [55] |
|  | *gdh* | GDHeF | TCAACGTYAAYCGYGGYTTCCGT | [56] |
|  |  | GDHiF | CAGTACACCTCYGCTCTCGG | [56] |
|  |  | GDHiR | GTTRTCCTTGCACATCTCC | [56] |
|  | *bg* | G7_F | AAGCCCGACGACCTCACCCGCAGTGC | [57] |
|  |  | G759_R | GAGGCCGCCCTGGATCTTCGAGACGAC | [57] |
|  |  | G99_F | GAACGAACGAGATCGAGGTCCG | [57] |
|  |  | G609_R | CTCGACGAGCTTCGTGTT | [57] |
|  | *tpi* | AL3543 | AAATIATGCCTGCTCGTCG | [58] |
|  |  | AL3546 | CAAACCTTITCCGCAAACC | [58] |
|  |  | AL3544 | CCCTTCATCGGIGGTAACTT | [58] |
|  |  | AL3545 | GTGGCCACCACICCCGTGCC | [58] |
| *Cryptosporidium* spp. | *ssu* rRNA | CR-P1 | CAGGGAGGTAGTGACAAGAA | [59] |
|  |  | CR-P2 | TCAGCCTTGCGACCATACTC | [59] |
|  |  | CR-P3 | ATTGGAGGGCAAGTCTGGTG | [59] |
|  |  | CPB-DIAGR | TAAGGTGCTGAAGGAGTAAGG | [59] |
| *Cryptosporidium hominis*/*parvum* | *gp60* | CR-P1 | CAGGGAGGTAGTGACAAGAA | [60] |
|  |  | CR-P2 | TCAGCCTTGCGACCATACTC | [60] |
|  |  | CR-P3 | ATTGGAGGGCAAGTCTGGTG | [60] |
|  |  | CPB-DIAGR | TAAGGTGCTGAAGGAGTAAGG | [60] |
| *Cryptosporidium viatorum* | *gp60* | CviatF2 | TTCATTCTGACCCCTTCATAG | [45] |
|  |  | CviatR5 | GTCTCCTGAATCTCTGCTTACTC | [45] |
|  |  | CviatF3 | GAGATTGTCACTCATCATCGTAC | [45] |
|  |  | CviatR8 | CTACACGTAAAATAATTCGCGAC | [45] |
| *Blastocystis* spp. | *ssu* rRNA | BhRDr | GAGCTTTTTAACTGCAACAACG | [61] |
|  |  | RD5 | ATCTGGTTGATCCTGCCAGT | [61] |

*bg*: β-giardin; *gdh*: Glutamate dehydrogenase; *ssu* rRNA: Small subunit ribosomal RNA; *tpi*: Triose phosphate isomerase.
